# Immersive virtual reality during gait rehabilitation increases walking speed and motivation: a usability evaluation with healthy participants and patients with multiple sclerosis and stroke

**DOI:** 10.1186/s12984-021-00848-w

**Published:** 2021-04-22

**Authors:** Carla Winter, Florian Kern, Dominik Gall, Marc Erich Latoschik, Paul Pauli, Ivo Käthner

**Affiliations:** 1 Department of Psychology I, Biological Psychology, Clinical Psychology And Psychotherapy, University of Würzburg, Marcusstraße 9–11, 97070 Würzburg, Germany; 2 Human-Computer Interaction, University of Würzburg, Würzburg, Germany; 3 Center of Mental Health, Medical Faculty, University of Würzburg, Würzburg, Germany

**Keywords:** Rehabilitation, Gait disorder, Virtual reality, Multiple sclerosis, Stroke, Head-mounted display, Motivation

## Abstract

**Background:**

The rehabilitation of gait disorders in patients with multiple sclerosis (MS) and stroke is often based on conventional treadmill training. Virtual reality (VR)-based treadmill training can increase motivation and improve therapy outcomes. The present study evaluated an immersive virtual reality application (using a head-mounted display, HMD) for gait rehabilitation with patients to (1) demonstrate its feasibility and acceptance and to (2) compare its short-term effects to a semi-immersive presentation (using a monitor) and a conventional treadmill training without VR to assess the usability of both systems and estimate the effects on walking speed and motivation.

**Methods:**

In a within-subjects study design, 36 healthy participants and 14 persons with MS or stroke participated in each of the three experimental conditions (VR via HMD, VR via monitor, treadmill training without VR).

**Results:**

For both groups, the walking speed in the HMD condition was higher than in treadmill training without VR and in the monitor condition. Healthy participants reported a higher motivation after the HMD condition as compared with the other conditions. Importantly, no side effects in the sense of simulator sickness occurred and usability ratings were high. No increases in heart rate were observed following the VR conditions. Presence ratings were higher for the HMD condition compared with the monitor condition for both user groups. Most of the healthy study participants (89%) and patients (71%) preferred the HMD-based training among the three conditions and most patients could imagine using it more frequently.

**Conclusions:**

For the first time, the present study evaluated the usability of an immersive VR system for gait rehabilitation in a direct comparison with a semi-immersive system and a conventional training without VR with healthy participants and patients. The study demonstrated the feasibility of combining a treadmill training with immersive VR. Due to its high usability and low side effects, it might be particularly suited for patients to improve training motivation and training outcome e. g. the walking speed compared with treadmill training using no or only semi-immersive VR. Immersive VR systems still require specific technical setup procedures. This should be taken into account for specific clinical use-cases during a cost–benefit assessment.

**Supplementary Information:**

The online version contains supplementary material available at 10.1186/s12984-021-00848-w.

## Background

The prevalence of gait disorders resulting from neurological disorders such as multiple sclerosis (MS) and stroke is high [1–3] and expected to further increase in the coming years due to the demographic change [4, 5]. Most patients with MS suffer from walking impairments as their main problem [6]. Impaired walking can occur early in the course of MS [7] and 15 years after diagnosis, 40% of patients require walking aids [8]. Of the two thirds of people who survive a stroke, more than 60% suffer from impaired walking abilities after an acute infarction and require gait rehabilitation [9–11].

Gait disorders can result from general muscle weakness, paresthesia, cerebellar coordination problems, general fatigue or a disorder of central gait control [12]. Typical manifestations are reduced stride length or walking speed and loss of balance control [13]. The walking limitations cause severe restraints in daily life, result in an increased risk of falling [14, 15], and a reduced quality of life for those affected [16, 17]. To maintain the patient's independence as long as possible, a gait disorder must be managed early and consistently. Standard treatments are physical therapy or exercise therapy [18, 19]. An essential element of these forms of therapy is treadmill training [20, 21]. If required, it can be combined with body-weight-supported systems [22] or robotic assistance such as in active orthoses [23, 24]. Regular treadmill training can reduce motor deficits of the lower limbs and significantly improve the patients’ walking abilities [25]. In patients with stroke, it can also enhance gait symmetry, gait uniformity and walking speed [26, 27]. However, the training structure is based on regularity and repetition [20, 25, 28]. For patients with gait disorders, who may depend on lifelong training, this training structure offers limited variety and could lead to low motivation and a lack of adherence in the long term. Treadmill training can be combined with virtual reality (VR) to increase its efficacy and the patients’ motivation, as demonstrated in several studies [28–33]. For instance, a recent study complemented a robot-assisted gait training with a semi-immersive VR presentation via a monitor [29]. At the end of an eight-week training, a 20% improvement in gait and balance was demonstrated for patients with MS. Importantly, the training with VR had positive effects on the patients' attitudes and coping strategies for dealing with their disease. In a randomized controlled trial with patients with MS comparing conventional with VR-based treadmill training, both groups improved walking endurance and speed [30]. Persons with gait disorders caused by a stroke can also benefit from regular, VR-supported gait training [31, 34]. In this group of patients, the cause of an uncertain gait pattern is usually a balance impairment [35]. Targeted treadmill training in VR can help patients regain their balance and reduce their risk of falling [36]. Most previous studies with patients have used either semi-immersive or immersive VR systems [33]. Until now, no study has conducted a direct comparison of an immersive and a semi-immersive VR based treadmill training with patients with stroke and MS. For the current study, a novel VR-based treadmill training was implemented and its feasibility tested with healthy participants and patients with stroke and MS with gait disorders. The virtual scenario that was created for this study aimed at increasing motivation with an engaging storyline and gamification elements to foster the experience of relatedness, competence and autonomy [37, 38]. We followed a well-established development regime in medical-oriented human–computer systems. To ensure that the immersive VR treadmill training increases motivation and has no negative side effects, we conducted a first usability study with healthy participants prior to the current study [38]. In that study, the immersive VR treadmill training was compared with a conventional treadmill training without VR. For the current study, a semi-immersive VR treadmill training was added as a further control condition. This is essential to assess the advantages and disadvantages of an immersive and semi-immersive VR system, respectively—in particular, in light of the increased effort needed to setup an immersive system and possible side effects previously reported, such as simulator sickness [39]. Thus, all participants took part in three conditions in which they tested an immersive VR system (HMD), a semi-immersive VR system (presented via a monitor) and conventional treadmill training (electric treadmill with manual speed adjustment) without additional VR.

The aim of the study was to evaluate an immersive VR application for supervised gait rehabilitation of patients with MS or stroke, to test its feasibility and acceptance and to compare its effects to those of a semi-immersive application and to a conventional treadmill training. First, healthy people participated followed by persons with MS or stroke. For both studies, walking speed served as an indicator of the short-term effectiveness of the systems. Heart rate was assessed as additional objective measure before and after each condition. Furthermore, the usability of the system was systematically evaluated by the participants with questionnaires and rating scales and potential side effects, mood and motivation were assessed.

## Methods

### Participants

The studies with healthy participants and patients were conducted consecutively. N = 36 students participated in the first study (26 female, *M* = 22 years, *SD* = 3.7, range 19—39). They were compensated with course credit. None of the students had cardiovascular, neurological or psychiatric disorders.

N = 14 patients with neurological disorders (n = 10 MS, n = 4 stroke) participated in the second study (*M* = 52.6 years, *SD* = 7.5). Additional file 1: Table S1 lists patient characteristics. Patients’ autonomy and independence when walking on level ground were checked in advance by phone interview to ensure suitability for treadmill training without body-weight support (Expanded Disability Status Score (EDSS) < 6 [40] and Functional Ambulation Categories-Score ≥ 4 [41]). Exclusion criteria for patients with MS were an acute relapse or cortisone therapy in the 30 days prior to the study. Patients had normal or corrected-to-normal vision (and no nystagmus or diplopia) and were free from cardiorespiratory instability, epilepsy, spasticity, sensory ataxia, severe muscle weakness, paroxysmal vertigo and psychosis. The exclusion criteria were checked via self-reports of the patients. Due to a weight limitation of the treadmill, only patients with a weight below 150 kg could participate in the study.

The study was conducted in accordance with the Declaration of Helsinki. The Ethical Review Board of the Medical Faculty of the University of Würzburg approved the study protocol.

### Outcome measures

#### Short-term effects

The average walking speed during walking on a treadmill served as an indicator of the short-term effectiveness of the different treadmill training systems (with and without VR). Since short-term effects are likely to lead to an improved walking speed in daily life following long-term training, they are an initial indicator of the effectiveness of the systems. As an additional objective measure, the heart rate of the participants in the three experimental conditions was measured electronically using the treadmill data. The heart rate was measured for one minute before and after each condition using a pulse sensor attached to the wrists of the participants, because we were interested in changes in heart rate caused by the individual conditions. We expected an increase in heart rate caused by anxiety or stress in the immersive VR condition due to the novelty of the experience for the participants.

#### Further usability assessments

The following rating scales and questionnaires served to further evaluate the usability of the two VR conditions and the treadmill training without VR. The Borg Scale [42] and the Raw Task Load Index (RTLX), a short version of the NASA Task Load Index [43], served to estimate the subjective workload after each condition. The participants were asked to rate their mood and motivation verbally after each experimental condition on numerical rating scale from 0 (“very bad”) to 10 (“very good”). Mood and motivation of the healthy participants were assessed at the beginning of the study and after each condition. The patients' mood and motivation were assessed before and after each condition. After each condition, participants were asked how quickly they felt time passed on the treadmill on a scale from 0 (“very slow”) to 10 (“very fast”). After the VR conditions, participants were asked to rate their virtual experience on different scales ranging from 0 to 10 (see Tables 1 and 2). They were asked to rate their sense of presence in the virtual environment, how motivating they perceived the virtual world and how much attention they paid to their natural gait, while being immersed in VR.

**Table 1:** Comparisons of subjective ratings between the treadmill conditions in the study with healthy participants

Measure	Subscale	No VR^a^	Semi-immersive VR^a^ (monitor)	Immersive VR^a^ (HMD)	*p*	Cohen’s *d*	*t*	*F*
Mood^b^		7.00 (1.66)	7.61 (1.25)	8.39 (1.15)	< .001*			21.38
Motivation^b^		6.89 (2.29)	7.28 (1.78)	8.42 (1.38)	< .001*			11.98
IMI	Interest/Enjoyment		4.63 (1.15)	5.78 (0.96)	< .001*	1.29	− 7.73	
	Perceived Competence		4.81 (0.85)	5.18 (0.78)	.002*	0.42	− 3.42	
	Effort/ Importance		3.89 (1.29)	4.24 (1.20)	.041*	0.36	− 2.12	
	Pressure/ Tension		2.01 (0.69)	1.84 (0.84)	.113	0.27	1.63	
Sense of time^c^		4.25 (2.38)	6.64 (1.57)	7.89 (1.33)	< .001 (GG− corrected)*			52.15
VR-questions^d^	“The virtual environment was motivating for me”		7.5 (1.65)	8.53 (1.36)	< .001*	0.78	− 4.67	
	“I felt present in the virtual environment”		5.72 (1.95)	8.06 (1.37)	< .001*	1.10	− 6.59	
	“I paid attention to my natural gait during the treadmill training”		5.64 (2.38)	5.28 (2.17)	.344	0.16	0.96	
IPQ	Spatial Presence		− 0.58 (0.88)	1.11 (0.53)	< .001*	2.00	− 12.01	
	Involvement		− 0.39 (0.78)	0.78 (0.56)	< .001*	1.43	− 8.58	
	Experienced Realism		− 1.31 (0.78)	− 0.24 (1.71)	< .001*	1.06	− 6.33	
	General		− 1.00 (1.71)	1.47 (1.21)	< .001*	1.37	− 8.26	
SSQ	Nausea		10.34 (11.49)	15.37 (11.47)	.014*	0.43	− 2.57	
	Oculomotor		12.63 (14.61)	12.84 (19.58)	.895	0.02	− .13	
	Disorientation		10.83 (17.00)	17.40 (26.07)	.061	0.32	− 1.94	
	Total		13.19 (13.34)	17.04 (18.46)	.068	0.31	− 1.88	
RTLX	Mental Demand	3.19 (4.95)	7.78 (7.79)	12.08 (11.17)	< .001*			15.75
	Physical Demand	10.69 (9.80)	13.61 (12.91)	15.00 (14.19)	.041*			3.35
	Temporal Demand	6.53 (7.82)	10.83 (14.12)	9.44 (15.30)	.156			1.91
	Effort	17.36 (23.89)	15.14 (21.40)	15.69 (26.68)	.752			.29
	Performance	9.72 (9.02)	13.19 (13.48)	12.50 (14.47)	.080			2.63
	Frustration	6.67 (8.62)	6.11 (9.42)	3.19 (7.09)	.083			2.58
Borg Scale		8.47 (1.63)	9.08 (1.81)	8.92 (2.17)	.207			1.61
SUS	Total		83.68 (9.36)	83.75 (10.03)	.965	< 0.01	− .05	

*IMI* Intrinsic Motivation Inventory, *IPQ* Igroup Presence Questionnaire, *RTLX* Raw NASA-Task Load Index, Raw National Aeronautics and Space Administration-Task Load Index, *SSQ* Simulator Sickness Questionnaire, *SUS* System Usability Scale

^a^Mean (SD)

^b^Range 0 (“Very bad”)—10 (“Very good”)

^c^Range 0 (“Very slow”)—10 (“Very fast”)

^d^Range 0 (“Fully disagree”)—10 (“Fully agree”)

*Significant *p*-values (*p* < .05) of main effects are marked with asterisks

**Table 2:** Comparisons of subjective ratings between the treadmill conditions in the study with patients

Measure	Subscale	No VR^a^	Semi-immersive VR^a^ (monitor)	Immersive VR^a^ (HMD)	*p*	Cohen’s *d*	*t*	*F*
Mood^b^		8.29 (1.20)	8.38 (1.61)	8.71 (1.49)	.580			0.56
Motivation^b^		8.36 (1.15)	8.08 (1.32)^e^	8.50 (2.31)	.618			0.49
IMI	Interest/Enjoyment		4.38 (0.89)	4.76 (0.49)	.212	0.35	− 1.31	
	Perceived Competence		4.36 (0.96)	4.57 (1.33)	.336	− 0.27	− 1	
	Effort/ Importance		3.26 (1.41)	3.47 (1.10)	.637	0.13	− 0.48	
	Pressure/ Tension		3.34 (0.56)	3.43 (0.51)	.708	0.10	− 0.38	
Sense of time^c^		8.43 (1.99)	8.00 (2.88)	8.00 (1.92)	.800			.23
VR-questions^d^	“The virtual environment was motivating for me”		7.50 (2.93)	8.43 (2.31)	.202	0.36	− 1.34	
	“I felt present in the virtual environment”		5.86 (3.11)	7.64 (2.10)	.032*	0.64	− 2.41	
	“I paid attention to my natural gait during the treadmill training”	4.50 (3.61)	4.79 (3.04)	5.93 (3.67)	.281			1.33
IPQ	Spatial Presence		− 0.44 (1.44)	1.03 (0.94)	.008*	0.83	− 3.12	
	Involvement		− 0.34 (0.85)	0.63 (0.84)	.009*	0.81	− 3.06	
	Experienced Realism		− 0.70 (1.01)	0.07 (1.15)	.001*	1.18	− 4.39	
	General		− 1.21 (2.01)	0.79 (2.12)	.003*	0.98	− 3.67	
SSQ^f^	Nausea		7.50 (8.52)	10.90 (12.88)				
	Oculomotor		10.29 (14.45)	11.37 (11.80)				
	Disorientation		5.97 (10.52)	10.94 (14.63)				
	Total		9.62 (12.16)	12.82 (11.98)				
RTLX	Mental Demand	5.71 (8.29)	10.00 (15.57)	17.14 (18.37)	.014*			5.06
	Physical Demand	10.36 (13.79)	20.71 (17.31)	21.79 (20.53)	.030*			4.05
	Temporal Demand	6.43 (12.16)	12.86 (15.53)	11.43 (11.10)	.057			3.20
	Effort	15.71 (17.64)	18.21 (20.90)	19.29 (21.91)	.737			0.31
	Performance	11.07 (13.47)	21.43 (21.16)	21.43 (22.82)	.114			2.36
	Frustration	2.14 (5.79)	5.36 (8.87)	4.29 (7.30)	.295 (GG-corrected)			1.23
Borg Scale		8.57 (1.60)	9.79 (2.55)	9.71 (2.73)	.075			2.86
SUS	Total		84.29 (14.29)	83.21 (18.28)	.830	.06	.22	

*IMI* Intrinsic Motivation Inventory, *IPQ* Igroup Presence Questionnaire, *RTLX* Raw NASA-Task Load Index, Raw National Aeronautics and Space Administration-Task Load Index, *SSQ* Simulator Sickness Questionnaire, *SUS* System Usability Scale

^a^Mean (SD)

^b^Range 0 (“Very bad”)—10 (“Very good”). Mood and motivation recorded after the treadmill conditions

^c^Range 0 (“Very slow”)—10 (“Very fast”)

^d^Range 0 (“Fully disagree”)—10 (“Fully agree”)

^e^n = 13

^f^Measure results after the treadmill conditions; no *p*-values for the post-measurement are listed here, since the focus of the evaluation was on the difference between the pre- and post-measurement, not between the post-measurements as in the study with the healthy participants

*Significant *p*-values (*p* < .05) of main effects are marked with asterisks

Intrinsic motivation was assessed using the Intrinsic Motivation Inventory (IMI) [44] and the sense of presence with the Igroup Presence Questionnaire (IPQ) [45]. Possible side effects of VR were assessed with the Simulator Sickness Questionnaire (SSQ) [46]. With this 16-item self-report measure, participants indicated the severity of individual symptoms. The questionnaire yields a total score and subscores for nausea, disorientation and oculomotor symptoms. Patients filled in the questionnaire before and after each VR-condition, while healthy participants filled in the questionnaire only after each condition. In order to investigate further aspects of the usability of the VR system the participants filled in the Equipment and Display Questionnaire (EDQ) [47]. It includes questions about three categories: the occurrence of physical discomfort due to HMD use, VR-related postural difficulties and problems with the visual display, such as distortion or insufficient resolution of the display. As another usability measure, the widely applied System Usability Scale (SUS) [48] was administered. It yields a total score ranging from 0 to 100, with higher values indicating higher usability.

#### Acceptance and satisfaction

At the end of the study, participants were asked which treadmill condition they liked best or least and which system they would like to use more often in the future. In addition, open-ended questions allowed them to comment on their experiences during testing and provide feedback and suggestions for improvement.

### VR-System

The treadmills for healthy participants (cardiostrong TR-30, Sport Tiedje GmbH, Schleswig, Germany) and for patients (mercury®med, h/p/cosmos sports & medical GmbH) both allowed for manual speed adjustments in 0.1 km/h steps. Patients wore safety belts to prevent falls. Before each condition, participants were instructed to hold onto the side handles and to walk at a comfortable walking speed, as they would during their daily walks. To achieve this individual speed, they were reminded before each condition to use the integrated buttons on the side handles to continuously adjust the speed of the treadmill during each treadmill condition.

For the semi-immersive VR condition, a 55″ television screen was positioned in front of the treadmill. In the immersive VR condition, the virtual environment was presented via a head-mounted display (HTC Vive, HTC Corp., New Taipei City, Taiwan). In this condition, the HTC Vive trackers were attached to participants’ shoes to allow synchronous foot movements of the avatar in the virtual environment. In both VR conditions, sounds of the virtual environment were played via circumaural headphones.

### VR-Environment

**Fig. 1:**
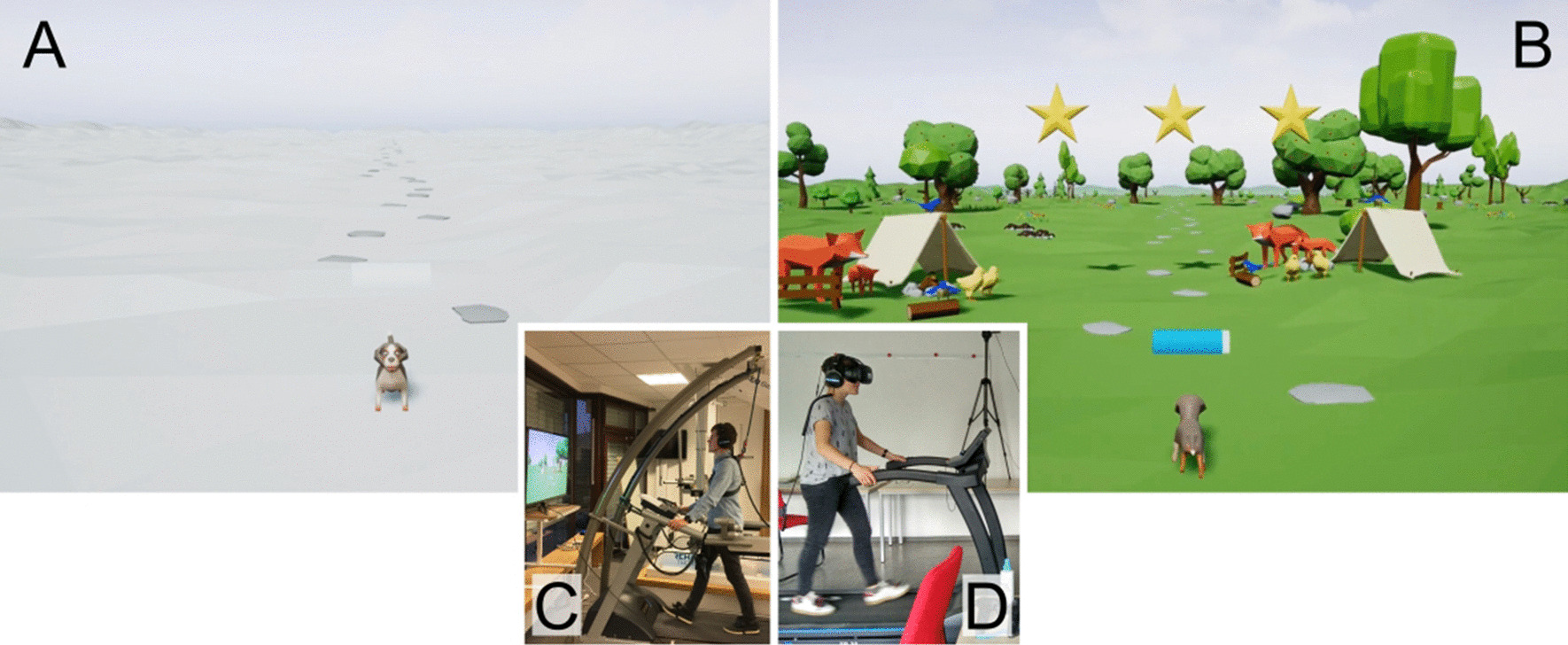
Screenshots of the virtual environment with overlays showing the study setup. At the beginning of the virtual scenario, the users meet a small virtual dog in a lifeless, deserted environment (A). By walking on the treadmill, the users can help rebuild its habitat, which continuously gets more fertile and colorful (B). With every star they collect on their way, the progress bar fills up further. Overlays depict the treadmill setup for patients (in the monitor condition, C) and the setup for healthy participants in the HMD condition (D)

For this study, a VR scenario named "Homecoming" was implemented using the Unreal Engine (for details see Kern et al. [38]). The VR scenario is displayed from a first-person perspective and aimed at increasing training motivation with an engaging storyline and gamification elements. At the beginning of the scenario, the users see a small path leading through a grey, deserted environment. A virtual companion personified as a dog explains the task to the users (see Fig. 1). They can help rebuild his world by walking on the path. When walking on the treadmill, the world gets continuously more fertile and colorful until it is completely rebuilt. For completing certain distances, users are awarded virtual stars as achievements in the virtual environment that are accompanied by positive comments of the virtual companion.

In the immersive VR condition, virtual shoes are displayed at the position of the real feet. In addition, safety features were implemented: When participants look down, a camera image from the real environment is superimposed on the virtual environment and superimposed green or red arrows serve as warning signals if the user is standing too far forward or backward on the treadmill, respectively.

To avoid frustration and ensure that all patients reach the end of the path, the length of the path was individually adjusted based on their average walking speed in the condition without VR. The length of the path was adapted so that the finish could be reached within 7.5 min.

### Procedure

**Fig. 2:**
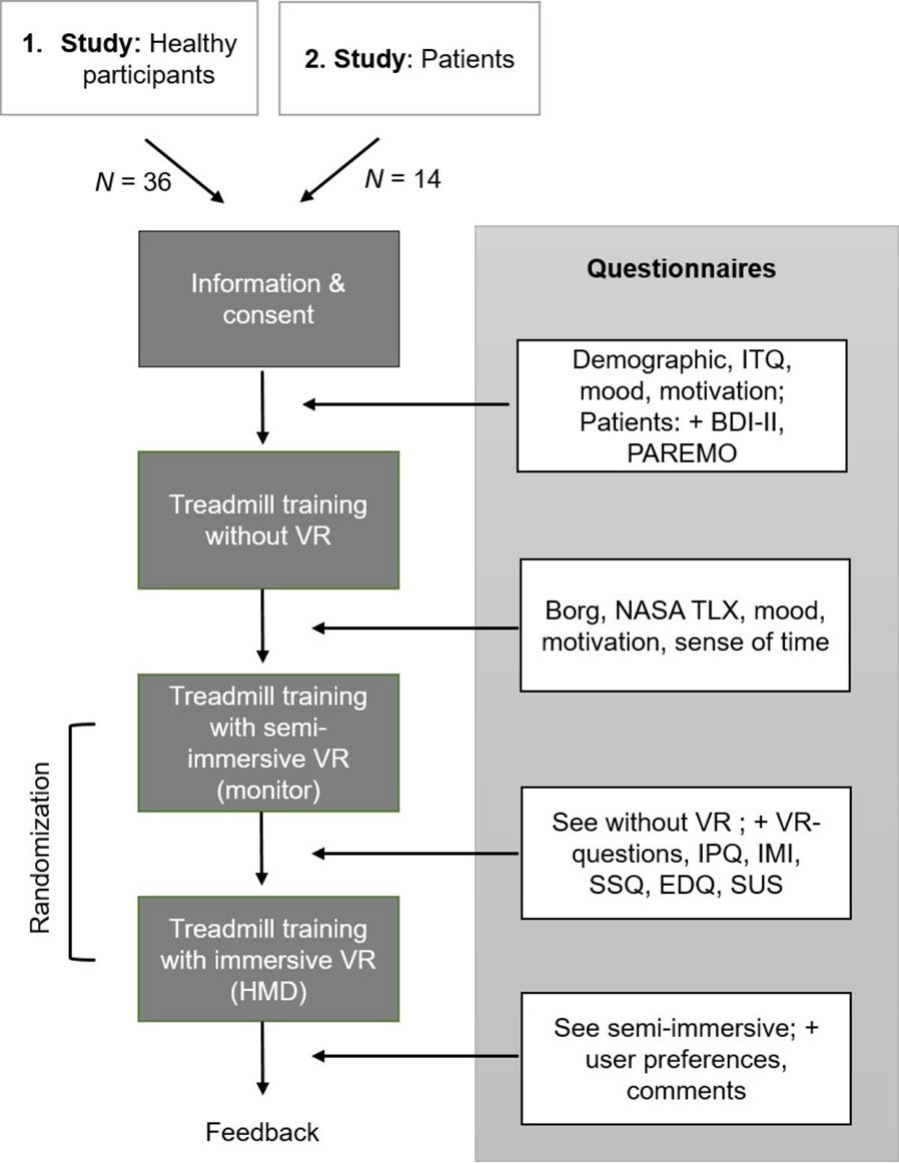
Study procedure. The study followed a within-subject design. The study consisted of three treadmill conditions which differed in the type of VR-presentation (no VR, semi-immersive VR and immersive VR). Between the conditions, participants answered questionnaires about their experience. Healthy participants completed the treadmill conditions on a single day (in pseudo-randomized order) and patients on two different days to avoid fatigue effects. *VR* Virtual Reality, *HMD* Head-Mounted-Display, *ITQ* Immersive Tendencies Questionnaire, *BDI-II* Beck Depression Inventory-II, *PAREMO* Patient Questionnaire for assessing Rehabilitation Motivation, *RTLX* Raw NASA-Task Load Index, Raw National Aeronautics and Space Administration-Task Load Index, *IPQ* Igroup Presence Questionnaire, *IMI* Intrinsic Motivation Inventory, *SSQ* Simulator Sickness Questionnaire, *EDQ* Equipment and Display Questionnaire, *SUS* System Usability Scale

Figure 2 depicts the experimental procedure of the study.

All participants took part in three conditions: No VR, semi-immersive VR (monitor), immersive VR (HMD). Treadmill training without VR was the first treadmill condition to be completed for both healthy participants and patients to become familiar with the treadmill and to establish a baseline walking speed for the VR conditions. Healthy participants underwent all conditions in one session with the two VR-based treadmill conditions in pseudo-randomized order so that the order of VR conditions was balanced across participants. Patients participated in two sessions on separate days. In the first session, all patients only took part in the treadmill training without VR. In the second session, all patients completed the two VR-based treadmill conditions also in pseudo-randomized order. Before and between the conditions, participants answered questionnaires. Each run was set to last approximately 7.5 min. The first run included the condition without VR and yielded a benchmark of the average speed of the participants for the other two runs. Based on this speed, the distance of the VR world was adjusted at the beginning so that the participants reach the finish in the VR environment in about 7.5 min. In all conditions, participants were instructed to walk on the treadmill at a comfortable walking pace. In order to achieve this, the participants were required to adjust the speed of the treadmill during training using the side handles of the treadmill. Depending on how they adjusted their speed, they reached the finish of the VR environment earlier or later. Participants did not receive feedback on how fast and how long they were walking on the treadmill. After reaching the finish line within the VR world, the treadmill was stopped by the researcher.

### Statistical analysis

Statistical analysis of the data was performed with SPSS (Version 23). To determine whether the type of treadmill training (no VR, semi-immersive VR, immersive VR) or the presentation order of VR in the VR conditions had an effect on the dependent variables such as walking speed and heart rate, repeated measures analyses of variance (rmANOVAs) were calculated. In case of significant main effects, pairwise post-hoc comparisons were conducted (with Bonferroni correction). To investigate effects between the two VR conditions, such as the different influence on presence, t-tests for related samples were applied. For all tests, the a-priori defined significance level was *p* < 0.05.

## Results

### Short-term effects: Walking speed and heart rate

**Fig. 3:**
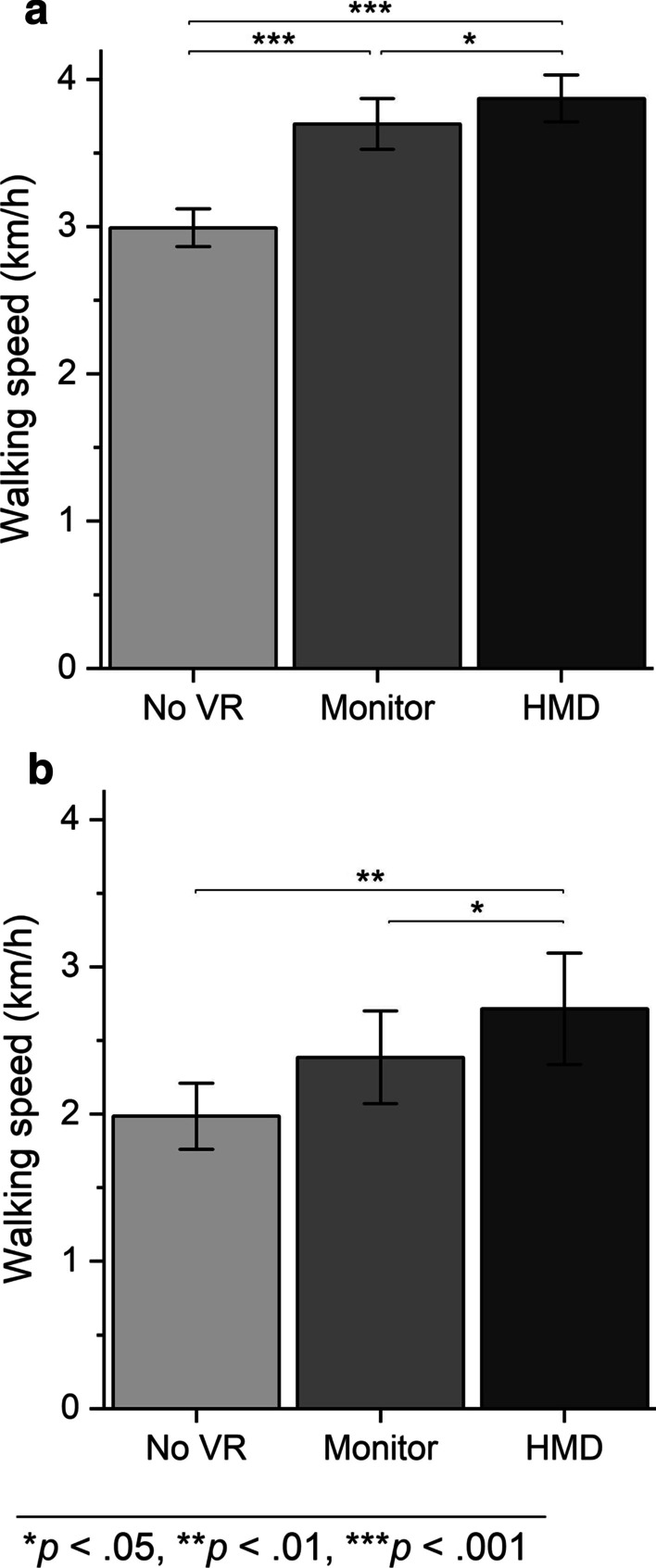
Average walking speed (± *SE*) for (**a**) healthy participants and (**b**) patients

Figure 3 depicts the average walking speed of the participants in the three experimental conditions.

For healthy participants, an effect of the type of treadmill training on the walking speed was detected, *F*_(1.59, 55.56)_ = 56.52, *p* < 0.001 (GG-corrected), partial η^*2*^ = 0.62. The walking speed in the HMD condition (*M* = 3.87 km/h, *SD* = 0.96) was higher than in the monitor condition (*M* = 3.70 km/h, *SD* = 1.03, *p* = 0.025) and higher than in the condition without VR (*M* = 2.99 km/h, *SD* = 0.77, *p* < 0.001). The walking speed in the monitor condition was higher as compared with the condition without VR (*p* < 0.001).

An effect of the type of treadmill training on walking speed was also observed in the patients, *F*_(2, 26)_ = 10.25, *p* < 0.001, partial η^*2*^ = 0.44. The walking speed was higher in the HMD condition (*M* = 2.71 km/h, *SD* = 1.42) than in the monitor condition (*M* = 2.39 km/h, *SD* = 1.18, *p* = 0.043) and higher than in the condition without VR (*M* = 1.99 km/h, *SD* = 0.84, *p* = 0.009). The walking speed in the monitor condition did not differ from the condition without VR (*p* = 0.068).

A repeated measures ANOVA revealed that there was neither an effect of presentation order on participants’ walking speed in the VR conditions (*F*_(1, 34)_ = 1.81, *p* = 0.188) nor an interaction effect between presentation type and presentation order (*F*_(1, 34)_ = 0.07, *p* = 0.791) for healthy participants. The ANOVA for patients yielded the same result. There was neither an effect of presentation order on patients’ walking speed in the VR conditions (*F*_(1, 12)_ = 0.64, *p* = 0.440) nor an interaction effect between presentation type and presentation order (*F*_(1, 12)_ = 0.13, *p* = 0.728).

Neither in the group of healthy participants (*F*_(2, 70)_ = 1.58, *p* = 0.214, partial *η*^*2*^ = 0.04) nor in the group of patients (*F*_(2, 26)_ = 1.21, *p* = 0.314, partial *η*^*2*^ = 0.09) did the type of treadmill training have any effect on the heart rate of the test participants (see Table 3).

**Table 3:** Means (and standard deviations) of the heart rates measured before and after each condition

	Condition	Pre	Post	Difference^a^	95% CI of the difference	*t*	*p*
Healthy participants	No VR	87 (9)	95 (9)	8 (13)	[4, 12]	3.7	.001*
	Semi-immersive VR	88 (10)	91 (11)	3 (13)	[8, − 1]	1.6	.122
	Immersive VR	89 (8)	91 (14)	2 (15)	[7, − 3]	0.8	.432
Patients	No VR	75 (17)	84 (18)	9 (18)	[− 1, 19]	1.9	.082
	Semi-immersive VR	74 (17)	77 (23)	3 (18)	[− 7, 13]	0.7	.524
	Immersive VR	79 (21)	77 (13)	− 2 (19)	[− 12, 9]	-0.3	.747

*CI* Confidence interval

^a^Difference = Heart rate post—Heart rate pre

*Significant *p*-values (*p* < .05) of main effects are marked with asterisks

### Further usability assessments

For all rating scales and questionnaires, scores for all conditions and test statistics for their comparisons are presented in Table 1 (for healthy participants) and in Table 2 (for patients). In the following sections, the results of pairwise comparisons are reported for the different outcome measures.

### VR-ratings

The results of the VR-rating scales are listed in Table 1 (for healthy participants) and Table 2 (for patients). Both groups stated that they felt more present in the VR world with the immersive VR presentation than with the semi-immersive presentation which served as a verification of the immersiveness manipulation (healthy participants: *p* < 0.001; patients *p* = 0.032). For the healthy participants, the virtual environment was more motivating when presented via the HMD than via the monitor (*p* < 0.001). Among patients, motivation was descriptively highest after the HMD condition, but no statistically significant differences were observed. There was no difference in the amount of attention paid to the natural gait during treadmill training in either group.

### Mood and motivation

The type of treadmill training had an influence on the mood of the healthy participants after the treadmill trainings (*p* < 0.001; see Table 1). The mood of the healthy participants was better after the HMD condition than after the monitor condition and higher than after the condition without VR (both *p* < 0.001). The mood after the monitor condition was better than in the condition without VR (*p* = 0.05). Likewise, the type of treadmill training influenced the motivation of the participants. The healthy participants reported higher motivation after the HMD condition than after the monitor condition (*p* < 0.001) and after the condition without VR (*p* < 0.001). The motivation in the monitor condition was not higher than in the condition without VR (*p* = 0.942).

In the patients, no significant differences between the patient mood before and after the treadmill trainings could be observed in any of the three conditions (*p* = 0.580; see Table 2). Considering the motivation, a difference in the monitor condition between pre- and post-measurement was detected (*p* = 0.032).

### Intrinsic motivation inventory (IMI)

In the study with healthy participants, a significant difference in IMI values was revealed between the monitor and HMD condition (see Table 1). The subscales *Interest* (*p* < 0.001), *Competence* (*p* = 0.002) and *Effort* (*p* = 0.041) had higher values in the HMD condition than in the monitor condition.

For patients, no differences between VR conditions for IMI scores were revealed, but descriptively there is a tendency similar to the results of the healthy participants (see Table 2).

### Sense of time

The type of treadmill training had an effect on the sense of time of the healthy participants (see Table 1). For healthy participants, subjectively, time elapsed faster in the HMD condition than in the monitor condition (*p* < 0.001) and faster than in the condition without VR (*p* = 0.001). The time passed faster in the monitor condition than in the condition without VR (*p* < 0.001). For patients, the type of treadmill training did not affect the sense of time (*p* = 0.800).

### Sense of presence (IGroup Presence Questionnaire)

For both, patients and healthy participants all values of the subscales of the IPQ (*Spatial Presence*, *Involvement*, *Experienced Realism* and the item assessing general presence) were higher in the HMD condition than in the monitor condition (see Tables 1 and 2). Agreement with the statement "I felt present in the virtual world." was higher in the HMD condition than in the monitor condition for healthy participants (*p* < 0.001) and for patients (*p* = 0.032).

### SSQ

In the healthy participants, no differences between the monitor and HMD condition were revealed for the *Total score* (*p* = 0.068) and the subscales *Oculomotor* (*p* = 0.895) and *Disorientation* (*p* = 0.061; see Table 1). For the subscale *Nausea*, however, higher values were reported in the HMD condition than in the monitor condition (*p* = 0.014). Neither in the monitor condition nor in the HMD condition did participants report very pronounced symptoms.

In the group of patients, there was no pre-post difference in SSQ total scores in either condition. In the monitor condition, significantly values were reported for all subscales after treadmill training, indicating reduced simulator sickness symptoms (*Nausea p* = 0.043, *Oculomotor p* = 0.015, *Disorientation p* = 0.019). In the HMD condition, a reduced score was observed in the subscale *Oculomotor* (*p* = 0.033). After the HMD condition, one patient reported considerable discomfort, another reported considerable dizziness with the respective items of the SSQ.

### Experienced workload

Neither in the group of healthy participants nor in the group of patients an effect of the type of treadmill training on the Borg scale was detected (see Tables 1 and 2).

The type of treadmill training did affect the subscale *Mental Demand* of the RTLX for both healthy participants (*p* < 0.001) and patients (*p* = 0.014). The mental load was higher in the HMD condition than in the condition without VR for patients (*p* = 0.039) and, in the healthy participants, higher than in the condition without VR (*p* < 0.001) and higher than in the monitor condition (*p* = 0.043). For patients, the type of treadmill training affected the subscale *Physical Demand* (*p* = 0.030). Post-hoc tests showed only trends towards significance for pairwise comparisons, such that *Physical Demand* in the HMD condition and the monitor condition was higher than in the condition without VR (*p* = 0.062 and *p* = 0.95, respectively). In both groups, the type of treadmill training did not affect the subscales *Temporal Demand*, *Effort*, *Performance* and *Frustration*.

### EDQ and SUS

The healthy participants mainly reported problems in the categories "physical discomfort" and "visual display" during HMD use. Nine participants (25%) felt uncomfortable and three (8%) criticized the quality of the display. In addition, 8% (n = 3) noticed flickering of the display, which was eliminated before the patient study.

None of the patients noticed any physical discomfort or flickering of the display during HMD use. Three patients (21%) had issues related to the quality of the visual display, mainly criticizing its level of detail and contrast.

For the SUS total score there were no differences between the HMD and monitor conditions in either study (see Tables 1 and 2). However, the mean total scores of 83 in both groups were very high for both semi-immersive and immersive VR systems, indicating high usability.

### Acceptance and satisfaction

In the group of healthy participants, 89% (n = 32) stated that they liked the treadmill training with the HMD best; 11% (n = 4) preferred the treadmill training with the monitor; 81% (n = 29) of the participants could imagine that patients with gait disorders would benefit from treadmill training with an HMD.

Among the patients, 71% (n = 10) liked the treadmill training with the HMD best. The same number would consider using the treadmill training with the HMD more often in the future and 64% (n = 9) are convinced that other patients with gait disorders could benefit from immersive VR-based treadmill training. Additional file 1: Table S2 provides an overview of the patients’ answers to the open-ended questions at the end of the study.

## Discussion

The present study demonstrated the feasibility and acceptance of an immersive virtual reality-based treadmill training as a rehabilitation method for neurological patients with gait disorders. The performance and usability of the immersive VR-based treadmill training was compared to a semi-immersive VR presentation (via monitor) and treadmill training without VR. The study demonstrated that the immersive presentation of a virtual scenario via an HMD leads to a higher walking speed of patients with MS and stroke than a semi-immersive VR presentation or a treadmill training without VR. The same holds true for healthy participants. The type of treadmill training did not affect the heart rate of the participants. For healthy participants, the mood and motivation of the participants were highest in the HMD condition, and, subjectively, the time elapsed faster. As expected, the sense of presence was higher in the immersive than in the semi-immersive condition for healthy participants and patients. As in our pilot study, no simulator sickness was observed (neither in the group of healthy participants nor in patients) [38]. The SSQ ratings were substantially lower compared with a recent study, in which individuals with Parkinson’s disease walked for 20 min on a treadmill while wearing a head-mounted display [49].

Walking speed is an important therapeutic outcome in patients with gait disorders and increasing walking speed is a common goal of gait rehabilitation [50, 51]. In our study, the patients walked faster in the VR-based treadmill conditions than in the one without VR. Since the mood was descriptively higher (however, not significantly for the patients) in these conditions, it can cautiously be reasoned that VR-supported training is more entertaining and exciting than training without VR—as demonstrated in several previous studies [29, 38]. In addition, the motivation of the healthy participants was higher after the VR conditions than after the treadmill training without VR, therefore, it is likely that increased mood and motivation contributed to the improved outcomes in the VR-assisted treadmill training and that patients will more likely perform a frequent training regime. As previously demonstrated in the study of Kern et al. (2019) the VR scenario implemented for the current study might be particularly suited to increase the users’ mood and motivation [38]. It includes gamification elements and the storyline is based on the self-determination theory of Ryan and Deci [37]. A novel element compared to previously implemented VR scenarios is the *social relatedness* aspect that was addressed in the current VR scenario with a virtual companion. Within the storyline, the user has the task of rebuilding the world for the virtual companion by walking on the treadmill. Thereby, the user can leave his patient role behind and take the role of an active helper instead. Further, the virtual companion provides encouraging comments, thereby contributing to increasing the users’ motivation. Although descriptively, motivation for patients was highest after the HMD condition, it was not statistically higher compared to the other conditions. Compared to motivation ratings of healthy participants, patients’ motivation ratings were already very high after the first treadmill session without VR (see Table 2). Hence, there was probably a ceiling effect. Individual user comments indicated that the VR was experienced as particularly motivating (see Additional file 1: Table S2).

### Immersive vs. semi-immersive VR-presentation

For the first time, the current study provides a direct comparison of immersive, semi-immersive VR-based gait rehabilitation and conventional gait rehabilitation with patients, which is essential to reveal the advantages and disadvantages of the respective methods. As expected, and in line with many previous studies [52, 53], healthy participants and patients alike reported a higher sense of presence in the virtual environment presented via an HMD compared to a semi-immersive presentation via a monitor. Due to the three-dimensional presentation and the panoramic view that covers the users’ field of view, it facilitates immersion in the virtual and distraction from the real world [52, 54]. Thus, the high degree of immersion in the HMD condition could be a reason for the higher intrinsic motivation of the healthy participants in this condition [55] and could explain that subjectively, time elapsed faster.

The treadmill training in all conditions was rated on the Borg scale with values below 10, which can be interpreted as "very easy" on a scale of 6 ("not exhausting at all") to 20 ("maximum exhausting") [42]. Values for healthy participants and patients were similar, even though for patients walking on level ground can be a challenge, depending on the severity of the disease and the form of the day [56] and all patients participated in a VR study (with an HMD) for the first time. Physical and mental demands were rated as low with the RTLX. For patients, however, mental demands were significantly higher in the HMD condition compared with the treadmill training without VR. And descriptively, these mental demands were highest in the HMD condition. For patients with neurological diseases the cognitive or visual system is sometimes also affected by the disease, which can have an influence on the ability to process information from the environment [57–59]. For those with severe cognitive and visual disturbances as well as for those with limited postural control and balance (e. g., caused by sensory deficits in the lower limbs), a semi-immersive presentation might be more suitable.

Importantly, however, the side effects of the immersive VR presentation were low for the vast majority of study participants. When using VR in neurological patients, visual or cognitive overload must always be avoided to prevent simulator sickness [60]. It can occur when there are discrepancies between the senses that provide information about the body’s motion and orientation [39]. It can be particularly problematic, when the virtual environment suggests movement, while the body is static [61, 62]. As an important measure to prevent simulator sickness in the current study, we synchronized the speed of visual motion in the virtual environment with the physical speed of the treadmill. Generally, measures should be undertaken to reduce the risk of simulator sickness. However, due to individual differences in the susceptibility to simulator sickness [39, 63], the decision whether a person receives immersive or semi-immersive VR gait rehabilitation should be made on a patient-by-patient basis.

Patients were not familiar using an HMD prior to this study. Since the VR-based treadmill training, in particular with the HMD, was a new experience for all participants, an increase in heart rate could be expected in the HMD condition, which could indicate anxiousness or stress. However, there were no pre-post differences for the VR conditions. Hence, the heart rate did not indicate an increase in sympathetic nervous system activity. The patients showed a slight increase in heart rate in the condition without VR, probably because this condition was always the first and no familiarization had taken place beforehand (see section “Limitations”). In addition to the unusual manipulation of several senses simultaneously while being fully immersed in a virtual world, the hardware (HMD) can also cause problems that limit the user's comfort and, in the worst case, contribute to simulator sickness. In particular, for persons wearing glasses, an HMD could exert pressure on certain regions of the head, such as the bridge of the nose, and result in a feeling of confinement. Nevertheless, in our study none of the patients experienced any physical discomfort during its use as assessed with the EDQ. The usability of the immersive VR-based treadmill training and the semi-immersive VR-based training were comparably high according to the results of the SUS. The total mean scores of 83 indicate that usability of the VR-based gait rehabilitation is “excellent” according to the ranges previously proposed [64]. When the VR setup is installed and calibrated, both the semi-immersive and the immersive VR system are as simple to use as an automated teller machine, for example [65]. Further, most of the patients stated that they liked the HMD-based treadmill training best and that they could imagine using it more often in the future.

### Limitations and outlook

This study revealed the feasibility and acceptance of an immersive VR-based treadmill training among a group of healthy users and patients with MS and stroke. Despite performance comparisons across only short sessions, a higher average walking speed was revealed for the HMD condition. This indicates that immersive VR might improve the therapeutic outcome in gait rehabilitation. Overground walking, however, was not assessed in the current study. While this study is an important first step, long-term use and training effects need to be evaluated next. Within our study we pooled findings from a heterogeneous and small group of patients with MS and stroke, hence the results are neither specific for the individual disorders not generalizable to all patients with MS or stroke or persons with neurological disorders. To increase the generalizability of the results a larger sample size is desirable. The immersive VR-based treadmill training might be less suited for neurological patients with visual or cognitive deficits. It remains to be tested if somatosensory deficits of the lower limbs, in particular proprioceptive deficits, are contraindications for the use of an HMD. Somatosensory deficits of the lower limbs are common in persons with multiple sclerosis and can negatively affect balance [66]. Occluding vision of the lower limbs (via the HMD) could further impair balance and walking as patients might rely on the visual information to maintain postural control.

Further, participants wore the HMD in the immersive VR condition for a relatively short amount of time (around 10 min). The usability for longer training sessions has yet to be assessed. In this study, the three experimental conditions were not preceded by a familiarization session on the treadmill and the treadmill condition without VR was always the first session for all participants. Therefore, the comparisons with the treadmill only condition (No VR) have to be interpreted with caution, because the results could potentially be confounded by acclimatization. Although the focus of the present study was on the comparison between the two VR treadmill conditions, it might be recommendable for future studies to precede the main training sessions by at least one, and in neurological patients possibly more familiarization sessions on the treadmill [67, 68].

### Further considerations for clinical use

Our study yielded promising results for the use of immersive VR in gait rehabilitation. However, in a clinical context and for future studies, additional aspects need to be considered that were not part of our evaluation, but could pose risks for the users. For instance, in light of the current Covid-19 pandemic adequate hygiene is crucial, and it takes a considerable amount of time to manually disinfect the HMD. However, first commercial disinfect devices based on ultraviolet light are now available. It is also necessary to accustom the participants to the HMD in order to prevent situational and cognitive overload. For patients with visual impairments, the HMD must be adjusted so that it does not generate any unpleasant or even painful sensations in combination with the patient's glasses. Further it should be mentioned that the setup of the HMD, in its current form, with its external sensors is more difficult than using a semi-immersive VR system (presentation via monitor) and requires additional efforts and skills.

The VR-based treadmill training presented can be extended to many other patient groups with gait disorders such as orthopedic patients or persons with Parkinson's disease, but for each patient group it must be re-evaluated whether and which type of VR presentation is best suited for the individual patient groups.

In any case, the effectiveness for health outcomes needs to be investigated within a clinical trial. The assessment of efficiency and satisfaction involving all end users (i. e. patients and caregivers) should be given a central role.

## Conclusions

The study demonstrated the feasibility and patients’ acceptance of a treadmill training with immersive VR via an HMD. Due to its excellent usability and low side effects, the system could serve as a valid alternative to conventional treadmill training in gait rehabilitation. It might be particularly suited for patients to improve training motivation and adherence, particularly if it is a fun training that captivates the user and is not perceived as mentally exhausting. Furthermore, the increased walking speed demonstrated in the current study suggests that it could lead to an improved training outcome compared with treadmill training using no or only semi-immersive VR. Despite these promising results, the decision, whether the advantages of immersive VR outweigh the risks associated with wearing an HMD should be based on a patient-by-patient basis and first and foremost on the will of the individual patient.

## Supplementary Information

**Additional file 1:** **Table S1.** Patient characteristics. **Table S2. **Patients' feedback on the study classified by thematic area.

## Data Availability

The datasets used and/or analyzed during the current study are available from the corresponding author on reasonable request.

## Abbreviations

HMD: Head-mounted display
IMI: Intrinsic Motivation Inventory
IPQ: IGroup Presence Questionnaire
MS: Multiple Sclerosis
RTLX: Raw Task Load Index
SSQ: Simulator Sickness Questionnaire
SUS: System Usability Scale
VR: Virtual Reality
