# Supplementary material for: Immersive virtual reality during gait rehabilitation increases walking speed and motivation: a usability evaluation with healthy participants and patients with multiple sclerosis and stroke
Source: J Neuroeng Rehabil. 2021 Apr 22;18:68. doi: 10.1186/s12984-021-00848-w (PMC8061882; doi:10.1186/s12984-021-00848-w)
Supplement: Supplementary file 1 — Additional file 1: Table S1. Patient characteristics. Table S2. Patients' feedback on the study classified by thematic area. [file 12984_2021_848_MOESM1_ESM.docx]

Supplementary Table S 1: Patient characteristics.

| Patient | Age | Sex | Diagnosis | Years since diagnosis | Years since onset of the gait disorder | Main gait impairment**^a^** | Fatigue**^b^** | BDI-II | PAREMO | | | | | |
| --- | --- | --- | --- | --- | --- | --- | --- | --- | --- | --- | --- | --- | --- | --- |
|  |  |  |  |  |  |  |  |  | Mental suffering | Physical restrictions | Social support | Readiness for change | Information status | Skepticism |
| 1 | 40 | F | MS | 4 | 4 | Walking insecurity, sensitivity impairments | Yes | 14 | 5 | 10 | 9 | 5 | 6 | 7 |
| 2 | 54 | F | MS | 22 | 22 | One-sided leg weakness, stumbling | Yes | 12 | 5 | 11 | 4 | 3 | 12 | 7 |
| 3 | 41 | M | Stroke | 10 | 10 | Claudication | No | 10 | 5 | 10 | 11 | 4 | 11 | 7 |
| 4 | 55 | M | MS | 28 | 5 | One-sided leg weakness | No | 4 | 4 | 7 | 12 | 3 | 12 | 8 |
| 5 | 65 | F | MS | 34 | 12 | Leg weakness | Yes | 5 | 3 | 11 | 7 | 4 | 12 | 4 |
| 6 | 42 | F | MS | 18 | 2 | One-sided leg weakness | Yes | 20 | 6 | 9 | 12 | 7 | 8 | 5 |
| 7 | 51 | F | Stroke | 12 | 12 | Claudication | No | 27 | 11 | 13 | 9 | 9 | 12 | 9 |
| 8 | 59 | M | MS | 20 | 20 | Stumbling | No | 5 | 6 | 12 | 9 | 6 | 11 | 8 |
| 9 | 59 | F | MS | 16 | 16 | Leg weakness, lack of leg control | No | 1 | 3 | 11 | 9 | 7 | 12 | 3 |
| 10 | 60 | M | Stroke | 8 | 6 | Sensitivity impairments in both legs | No | 22 | 5 | 16 | 9 | 11 | 10 | 8 |
| 11 | 52 | F | MS | 10 | 4 | Claudication | Yes | 7 | 3 | 12 | 14 | 5 | 11 | 7 |
| 12 | 56 | M | Stroke | 0 | 0 | Balance disturbance | No | 4 | 5 | 6 | 12 | 8 | 8 | 3 |
| 13 | 53 | M | MS | 6 | 5 | Claudication | Yes | 7 | 6 | 11 | 10 | 7 | 11 | 6 |
| 14 | 50 | F | MS | 30 | 6 | Claudication | No | 0 | 3 | 4 | 4 | 3 | 12 | 6 |

^a^self-reported; ^b^self-reported chronic fatigue syndrome. Abbreviations: BDI-II, Beck Depression Inventory-II; PAREMO, Patient Questionnaire for assessing Rehabilitation Motivation; MS, Multiple Sclerosis; F, female; M, male.

Supplementary Table S 2: Patients' feedback on the study classified by thematic area.

| Thematic area | Patient statements (“What I liked…”) | Patient statements (“What I did not like…”) |
| --- | --- | --- |
| Gait experience | "[...] that you can forget about your gait uncertainty via the VR-glasses and completely focus on the task at hand. You're in a virtual world and you're not thinking about your own troubles [with walking]." (Patient #2) |  |
| Training type | “Absolutely walking with the [VR-] glasses” (Patient #4)  “VR via VR glasses” (Patient #3)  “The treadmill workout with VR glasses because you don't focus on the environment, you just instinctively start walking.” (Patient #14) | "The second round without VR glasses, just using the monitor." (Patient #2)  “Walking in front of the display” (Patient #4)  "The conventional treadmill [training], boring and easily distractable" (Patient #14) |
| Image design | “Enjoyable images” (Patient #1) | "The not-yet fully developed virtual world." (Patient #13)  “The blur of the images” (Patient #1) |
| Story | “The motivation to help the animals.” (Patient #11)  “The subconscious motivation generated by the dog walking ahead.” (Patient #13) |  |
| Music | “Relaxing music” (Patient #1) |  |
